# Resistance training modalities: comparative analysis of effects on physical fitness, isokinetic muscle functions, and core muscle biomechanics

**DOI:** 10.3389/fphys.2024.1424216

**Published:** 2024-07-12

**Authors:** Peng Liu, Han Yuan, YunHang Lu, Zeng Gao

**Affiliations:** ^1^ College of Martial Arts and Traditional Ethnic Sports, Jilin Sport University, Changchun, China; ^2^ Department of Physical Education, Kyungpook National University, Daegu, Republic of Korea; ^3^ School of Physical Education and Sports Science, Soochow University, Suzhou, China; ^4^ School of Educational Studies, Universiti Sains Malaysia, Penang, Malaysia

**Keywords:** resistance training modalities, physical fitness, isokinetic muscle contraction, muscle biomechanics, pyramid set training

## Abstract

**Introduction:**

This study aimed to evaluate the effects of varied resistance training modalities on physical fitness components, body composition, maximal strength assessed by one-repetition maximum (1RM), isokinetic muscle functions of the shoulder and knee joints, and biomechanical properties of core muscles.

**Methods:**

Forty participants were randomly assigned to four groups: control group (CG, n = 10), compound set training group (CSG, n = 10), pyramid set training group (PSG, n = 10), and superset training group (SSG, n = 10). Excluding the CG, the other three groups underwent an 8-week resistance training program, three sessions per week, at 60%–80% of 1RM intensity for 60–90 min per session. Assessments included body composition, physical fitness components, 1RM, isokinetic muscle functions, and biomechanical properties (muscle frequency, stiffness, etc.) of the rectus abdominis and external oblique muscles.

**Results:**

The PSG demonstrated the most significant improvement in relative peak torque during isokinetic testing of the shoulder and knee joints. Compared to the CG, all exercise groups exhibited positive effects on back strength, sprint performance, 1RM, and core muscle biomechanics. Notably, the PSG showed superior enhancement in external oblique stiffness. However, no significant differences were observed among the exercise groups for rectus abdominis biomechanical properties.

**Discussion:**

Structured resistance training effectively improved maximal strength, functional performance, and core muscle biomechanics. The pyramidal training modality conferred specific benefits for isokinetic muscle functions and external oblique stiffness, suggesting its efficacy in enhancing force production capabilities and core stability.

## 1 Introduction

Resistance training is an effective exercise modality that can significantly enhance muscular strength, facilitate muscular hypertrophy, and improve overall physical fitness (Schoenfeld, 2010). This training approach elicits physiological stimuli, such as mechanical tension, muscle damage, and metabolic stress, through the manipulation of external loads, thereby initiating adaptive responses within the musculature (Florini, 1987). The primary variables in resistance training include training volume, intensity, rest intervals, and exercise order (Haff and Triplett, 2016). By strategically adjusting and combining these variables, various innovative training modalities can be derived. Pyramid training involves alternating between lighter and heavier intensities, such as a repetition scheme of 3-5-7-5-3, gradually increasing and subsequently decreasing the training load (Fleck and Kraemer, 2014; Cuevas-Aburto et al., 2020). Superset training entails performing two or more exercises targeting the same muscle group consecutively, such as pairing seated rows with pull-ups, augmenting metabolic stress and muscular fatigue (Robbins et al., 2010; Iversen et al., 2021). Compound training adheres to the traditional approach of training muscle groups in succession, selecting a primary exercise as the core and supplementing it with related assistance exercises, such as utilizing squats as the primary lower body exercise, complemented by calf raises and hip extensions (Haff and Triplett, 2016; Kraemer et al., 2002). Through various combinations of variables, these diverse training modalities can produce different outcomes, such as altering training stimuli and metabolic stress, thereby providing athletes with a range of training options. Judicious utilization of these modalities can help maintain novelty and sustained efficacy (Robbins et al., 2010; Pareja-Blanco et al., 2017).

Extant research indicates that different training modalities exert varying effects on strength development, physical fitness, and muscular characteristics. Certain studies have found that compound training can enhance one-repetition maximum (1RM) and overall muscular strength (Baz-Valle et al., 2019; Paoli et al., 2017), while pyramid training is more conducive to improving isometric muscular power (Mangine et al., 2015), and superset training can improve flexibility and reduce body fat percentage (Schoenfeld et al., 2014). However, the impacts of these training modalities on improving fundamental physical fitness, 1RM levels, and isometric muscular function remain contentious.

Non-invasive assessments of alterations in superficial muscle stiffness and tension can elucidate the pathological consequences of exercise and the efficacy of interventions (Dahmane et al., 2001; Marusiak et al., 2012). Previous investigations have substantiated the reliability of employing myotonometry (Myoton) to measure skeletal muscle stiffness and tension in diverse populations, such as healthy adults, individuals with Parkinson’s disease, and stroke survivors (Mullix et al., 2012; Marusiak et al., 2012; Chuang et al., 2013). Nevertheless, extant research has not comprehensively explored the effects of these training modalities on muscular mechanical properties, including tension, stiffness, and shape recovery ability.

Consequently, the present study endeavors to systematically compare prevalent resistance training modalities (pyramid sets, supersets, and compound sets) and comprehensively evaluate their respective impacts on physical fitness, isometric joint strength, and biomechanical properties of core muscle groups. The study will assess participants’ 1RM levels, isometric strength performance of limb joints, and employ non-invasive myotonometry techniques to quantify alterations in mechanical properties, such as muscle tension, stiffness, and shape recovery capacity, of core muscle groups. This research will not only augment existing knowledge but also provide empirical evidence to facilitate the development of individualized, goal-oriented training programs, optimizing training efficacy for diverse populations and objectives, thereby advancing theoretical and practical developments within related domains.

## 2 Materials and methods

### 2.1 Participants

This study employed a convenience sampling approach to recruit 40 male participants aged 18–30 years, each with a minimum of 6 months’ experience in resistance training. The participants were randomly assigned to four groups: Control Group (CG), Compound Set Group (CSG), Pyramid Set Group (PSG), and Superset Group (SSG), with 10 individuals in each group. Quality assurance measures were implemented to ensure participants met the following criteria: a) aged between 18 and 30 years, b) no positive response on the Physical Activity Readiness Questionnaire (PAR-Q), c) abstinence from substances that could influence the results (e.g., caffeine, creatine, steroids), and d) absence of musculoskeletal issues that might impede resistance exercise execution. This study received approval from the Institutional Review Board of Jeju National University (JJNU-IRB-2023-004). G*Power software (G*Power 3.1.9.7, University of Kiel, Kiel, Germany) was used to calculate the appropriate sample size. Additionally, the reference reported main effect size of 1.38 (Karasu et al., 2018), including a significance level α of 0.05 and a power 1-β of 0.95, calculated a sample size of 10 for each group. Table 1 presents the physical characteristics of the participants.

**TABLE 1:** Characteristics of participants.

	CG (n = 10)	CSG (n = 10)	PSG (n = 10)	SSG (n = 10)	*F*	*p*
Body height (cm)	174.1 ± 4.68	171.9 ± 4.43	173.2 ± 3.43	174.4 ± 4.3	0.70	0.557
Body weight (kg)	72.56 ± 5.58	72.89 ± 5.79	68.7 ± 6.23	72.53 ± 7.19	1.02	0.396
Age (yr)	19.7 ± 0.68	19.9 ± 0.74	19.9 ± 0.74	20.3 ± 0.82	1.14	0.346

CG, control group; CSG, compound set group; PSG, pyramid set group; SSG, super set group.

### 2.2 Exercise protocol

The 8-week resistance training intervention comprised three sessions per week, incorporating compound sets, pyramid sets, and superset routines. The initial 3 weeks served as an intensity adaptation phase, with training intensities individualized based on one 1RM test results. Each training session followed a structured protocol:

• Dynamic stretching: A Rated Perceived Exertion (RPE) scale of 9–11 was utilized to prepare the body for subsequent training.
• Running at 50%–70% of maximum heart rate: Lasted for 10 min to elevate heart rate and prepare the body for resistance training.
• Resistance training: including:
  1. Compound sets: Two exercises targeting the same muscle group performed consecutively, with a 1-min rest interval between sets.
  2. Pyramid sets: Traditional pyramid-style training, with progressive increments in weight and decrements in repetitions across sets.
  3. Superset: Two or more exercises targeting the same muscle group performed consecutively, without rest intervals.

Refer to the revised Table 2 for specific exercise details and loading parameters.

• Static stretching: Static stretching was performed for 5 min with an RPE rating of 6–7 as a cooldown exercise to alleviate muscle tension and minimize post-training discomfort.

**TABLE 2:** Resistance training program.

Session	Exercise						Time (min)
Warm-up	Dynamic stretching						10
	Running						
Type	Compound set Super set		Super set		Pyramid set		
Resistance training	Day 1	Day 2	Day 1	Day 2	Day 1	Day 2	40
	BP	SQ	BP	SQ	BP	SQ	
	IP	LP	ULP	SD	IP	LP	
	ULP	LG	IP	LE	ULP	LG	
	CR	LE	CR	LC	CR	SD	
	DSP	SD	DSP	LG	DSP	LE	
	SLR	LC	BLR	LP	SLR	LC	
Cool-down	Static stretching						5
Intensity	Compound set Super set		1∼3 weeks: 60% 1RM		Rest		120 s
			4∼6 weeks: 70% 1RM				
			7∼8 weeks: 80% 1RM				
	Pyramid set		1 set: 60% 1RM		Rest		90 s
			2∼3 set: 70% 1RM				
			4 set: 80% 1RM				

Note: BP, bench press; IP, incline push-up; ULP, under-grip lat pull down; CR, cable row; DSP, dumbbell shoulder press; SLR, side lateral raise; SQ, squat; LP, leg press; LG, lunge; LE, leg extension; SD, stiff deadlift; LC, leg curl; BLR, bent over lateral raise.

#### 2.2.1 Resistance training variables

The reporting of resistance training variables adhered to the recommendations by Coratella, G. (2022). The specific resistance training variables for each group were as follows:

• Control Group (CG): Performed traditional straight sets with three sets of eight to twelve repetitions at 60%–70% of 1RM for all exercises.
• Compound Set Group (CSG): Weeks 1–3: Three sets of eight to twelve repetitions at 60%–70% 1RM. Weeks 4–6: Three sets of six to eight repetitions at 70% 1RM. Weeks 7–8: Three sets of four to six repetitions at 80% 1RM. One-minute rest between compound sets.
• Pyramid Set Group (PSG): Day 1: One set at 60% 1RM, one set at 70% 1RM, one set at 80% 1RM. Day 2: One set at 80% 1RM, one set at 70% 1RM, one set at 60% 1RM. Ninety-second rest between pyramid sets.
• Superset Group (SSG): Weeks 1–3: Three sets of eight to twelve repetitions at 60-70% 1RM. Weeks 4–6: Three sets of six to eight repetitions at 70% 1RM. Weeks 7–8: Three sets of four to six repetitions at 80% 1RM. No rest between superset exercises.

### 2.3 Measurement procedures

#### 2.3.1 Body composition

The InBody 770 body composition analyzer (InBody, Seoul, South Korea) was utilized to measure height, weight, skeletal muscle mass, fat mass, body mass index (BMI), and body fat percentage. Participants refrained from food and fluid intake for 8 h before the measurement, wore light, form-fitting clothing, and removed any metallic objects. Body fat percentage was estimated using bioelectrical impedance analysis. BMI was calculated as weight (kg) divided by height (m) squared, while body fat percentage was estimated based on bioelectrical impedance analysis.

#### 2.3.2 Basic fitness tests

##### 2.3.2.1 Flexibility

Measure flexibility using the Takei TKK5111 sit-and-reach test. The participant sits with feet no more than 5 cm apart, then reaches forward with both hands toward the end of the device. Hold the position for 3 s and perform 2 trials, recording the maximum distance in centimeters (cm).

##### 2.3.2.2 Muscle strength

Assess dominant hand grip strength using the Takei TKK5101 digital grip strength meter and back strength using the Takei TKK5402 digital strength meter. Perform 2 trials and record the best score in kilograms (kg).

##### 2.3.2.3 40 m sprint

Measure time using the Casio OST-30W stopwatch. After one practice run, provide adequate rest. From the starting line, the participant runs at maximum speed to the 40 m mark, and the time is recorded when the chest crosses the finish line, to the nearest 0.01 s.

##### 2.3.2.4 Illinois agility test

Use the Casio OST-30W stopwatch to measure time on a standard 10 m × 5 m course. The participant starts on the signal, runs to the finish, and the best time of 2 trials is recorded in 0.01 s.

##### 2.3.2.5 Standing long jump

Stand with feet as close to the starting line as possible, then jump as far as possible. Record the distance from the start line to the nearest heel after 1 trial, with approximately 1 min of rest between the 2 measured jumps. Record the maximum distance in meters (m).

#### 2.3.3 One repetition maximum (1RM) of bench press, deadlift, and squat

The (1RM for the bench press, deadlift, and squat exercises was measured to assess the participants’ maximal strength. This assessment was conducted following the protocol recommended by the National Strength and Conditioning Association (NSCA) (Haff and Triplett, 2015). To ensure participant safety, at least two trained assistants were present to serve as spotters during the testing. Prior to the 1RM measurements, participants performed a standardized warm-up routine to prepare their musculoskeletal system and reduce the risk of injury. After the warm-up, participants attempted to lift the maximum weight they could for a single repetition on each of the three lifts. If a participant failed to complete a lift, the weight was immediately reduced by 5%, and the participant was given adequate rest before retrying the lift. This process continued until the participant’s 1RM was successfully determined for each exercise.

#### 2.3.4 Isokinetic shoulder and knee joint functions

To obtain accurate and reliable isokinetic peak torque data for the shoulder and knee joints, this study employed the Humac Norm isokinetic dynamometer (Humac Norm 776, CSMI, Boston, United States) and its Trunk Extension and Flexion (TEF) module. The isokinetic function of the shoulder and knee joints was assessed at an angular velocity of 60 degrees per second.

Prior to testing, participants performed three familiarization trials to acquaint themselves with the procedure and mitigate risks. Gravity correction was implemented during testing to eliminate the effect of gravity. The range of motion for shoulder testing was 30° internal rotation to 90° external rotation, measuring the isokinetic peak torque of the internal and external rotator muscle groups at 60°/s. The range of motion for knee testing was 0°–100° flexion-extension, measuring the isokinetic peak torque of the flexor and extensor muscle groups at 60°/s.

Each joint was tested six times bilaterally, with the average of the middle four results taken to enhance data reliability. To mitigate the influence of muscle fatigue, a 2-min interval was implemented between familiarization trials and formal testing, a 1-min interval between single sides, and a 2-min interval between both sides, with a 60-s recovery period after each test. The testing procedure was optimized to obtain high-quality data while minimizing the risk of harm to the participants.

#### 2.3.5 Measurement of muscle biomechanical properties

To evaluate the biomechanical performance of the trunk muscles, this study utilized the MyotonPRO (Myoton AS, Tallinn, Estonia) non-invasive contact soft tissue measurement device. This device is equipped with a three-axis digital accelerometer that can simultaneously obtain biomechanical parameters such as muscle tension and viscoelasticity by measuring the frequency decay and graphing the acceleration of natural tissue oscillations.

The measurements were conducted under controlled environmental conditions (limited resonant effects from noise and vibration, temperature 22°C–24°C, relative humidity 45%–60%). To ensure data accuracy, participants were instructed to remain relaxed during the measurement process to avoid producing unnecessary muscle tension. During measurement, the polycarbonate probe (3 mm diameter) was vertically applied to the center of the muscle belly to induce resonant vibrations. Muscle tension was represented by frequency (Hz), reflecting the intrinsic oscillation state without active contraction during baseline electromyography. The biomechanical parameters measured included dynamic stiffness (N/m) reflecting dynamic rigidity, logarithmic decrement reflecting vibration damping, stress relaxation time (ms), and creep reflecting viscoelasticity.

A multi-scan mode was employed, with the rectus abdominis (RA) and external oblique (EO) muscles measured to assess changes in the mechanical properties of the core muscles. The number of repetitions was set to five, with a tap time of 15 ms and a damping period of 0.8 s to ensure internal consistency. To improve the reliability of repeated measurements, all measurements before and after were performed by the same examiner. Prior to measurement, participants rested in a supine position for 20 min. During measurement, participants breathed naturally and maintained a stationary state without external force. Measurements were taken in the supine position, with the coefficient of variation (CV) observed on the MyotonPRO LCD monitor. Each site was measured twice; if CV > 3%, an additional measurement was taken, and the average of two measurements with CV ≤ 3% was used as the final result.

### 2.4 Statistical analysis

Statistical analyses were performed using IBM SPSS Statistics 22.0 (IBM Co., Armonk, NY, United States). Participants were randomly assigned to a control group (CG), compound set group (CSG), pyramid set group (PSG), or superset group (SSG). Pre-intervention and post-intervention data were collected to evaluate the effects of each training modality. All variables, encompassing pre-intervention and post-intervention values, were measured and recorded using SPSS 22.0. Means ± standard deviations (SDs) were calculated for each variable to describe the central tendency and dispersion of the data. The Shapiro-Wilk test (Razali, 2011) as employed to verify the normality of the dependent variables before and after the intervention, followed by Levene’s test to assess the homogeneity of variance.

Partial eta-squared (ηp^2^) was utilized as the effect size measure to quantify the magnitude of between-group differences. A two-way repeated measure analysis of variance (ANOVA) was conducted to evaluate the interaction between groups and time points for each variable, with *post hoc* analyses using Tukey’s correction. To compare the effects of different training programs on between-group differences, we employed ANOVA instead of an analysis of covariance (ANCOVA) with baseline as a covariate. This approach was chosen because the primary focus of this study was to assess the impact of different training programs on the changes in these variables, rather than accounting for individual differences. A significance level of *p* ≤ 0.05 was set to determine the statistical significance of the results.

## 3 Results

### 3.1 Body composition

The comparison of body composition among different resistance exercise sets (Table 3) revealed significant interactions between groups and time for weight, skeletal muscle mass, fat mass, and body fat percentage. However, according to *post hoc* tests, there were no significant differences among the groups in weight (*F = 1.089*, *p = .366*, ηp^2^ = 0.035), skeletal muscle mass (*F = 1.192*, *p = .327,* ηp^2^ = 0.038), fat mass (*F = .676*, *p = .573*, ηp^2^ = 0.038), and body fat percentage (*F = .724*, *p = .544*, ηp^2^ = 0.024).

**TABLE 3:** Changes in body composition according to the resistance training structure.

Variables	Group	Pre	Post		*F*	*p*	Tukey
Body weight (kg)	CG^a^	72.56 ± 5.58	73.22 ± 5.51	G	1.048	.383	
	CSG^b^	72.89 ± 5.79	72.94 ± 5.92	P	25.990	.001	
	PSG^c^	68.70 ± 6.23	68.85 ± 6.10	G × P	9.988	.001	
	SSG^d^	72.89 ± 7.19	72.60 ± 7.20				
Skeletal muscle mass (kg)	CG^a^	31.06 ± 2.80	31.12 ± 2.79	G	1.237	.311	
	CSG^b^	31.22 ± 3.48	31.70 ± 3.49	P	993.091	.001	
	PSG^c^	29.65 ± 2.84	30.34 ± 2.85	G × P	94.857	.001	
	SSG^d^	32.33 ± 2.95	32.81 ± 2.87				
Body fat mass (kg)	CG^a^	21.23 ± 4.05	21.84 ± 3.89	G	.499	.685	
	CSG^b^	22.06 ± 4.36	21.64 ± 4.48	P	17.626	.001	
	PSG^c^	20.79 ± 4.45	20.32 ± 4.37	G × P	39.487	.001	
	SSG^d^	20.07 ± 3.56	19.65 ± 3.36				
Body mass index (kg/m^2^)	CG^a^	23.92 ± 1.21	24.14 ± 1.19	G	2.143	.112	
	CSG^b^	24.67 ± 1.62	24.67 ± 1.64	P	16.562	.001	
	PSG^c^	22.88 ± 1.70	22.93 ± 1.67	G × P	7.950	.001	
	SSG^d^	23.81 ± 1.71	23.83 ± 1.76				
Percent body fat (%)	CG^a^	29.17 ± 4.57	29.76 ± 4.36	G	.618	.608	
	CSG^b^	30.07 ± 4.44	29.45 ± 4.58	P	58.737	.001	
	PSG^c^	30.15 ± 5.07	29.39 ± 5.04	G × P	48.613	.001	
	SSG^d^	27.73 ± 4.11	27.14 ± 3.90				

CG^a^, control group; CSG^b^, compound set group; PSG^c^, pyramid set group; SSG^d^, super set group; G, group; P, period; G × P, Group × Period.

### 3.2 Fundamental physical fitness

The analysis of fundamental physical fitness among various resistance exercise set compositions (Table 4) showed significant interactions between groups and time for sit-and-reach, grip strength, abdominal muscle strength, 40-m sprint, Illinois agility test, and standing long jump. However, significant differences were found in abdominal muscle strength (*F = 19.284*, *p = .001*, ηp^2^ = 0.395) and 40-m sprint (*F = 9.130*, *p = .001*, ηp^2^ = 0.245) among the groups in *post hoc* tests, while sit-and-reach (*F = .062*, *p = .980*, ηp^2^ = 0.002), grip strength (*F = 1.209*, *p = .320*, ηp^2^ = 0.039), Illinois agility test (*F = .478*, *p = .669*, ηp^2^ = 0.016) and standing long jump (*F = .177*, *p = .912*, ηp^2^ = 0.006) showed no significant differences. *Post hoc* tests indicated significant increases in abdominal muscle strength and 40-m sprint in CSG, PSG, and SSG, with no significant differences among the exercise groups.

**TABLE 4:** Changes in basic physical fitness according to the resistance training structure.

Variables	Group	Pre	Post		*F*	*p*	Tukey
Sit and reach (cm)	CG^a^	8.44 ± 2.42	8.50 ± 2.72	G	.074	.973	
	CSG^b^	6.98 ± 3.74	8.85 ± 3.14	P	24.625	.001	
	PSG^c^	7.98 ± 3.13	8.75 ± 3.31	G × P	7.134	.001	
	SSG^d^	8.08 ± 1.82	8.37 ± 1.83				
Grip strength (kg)	CG^a^	46.60 ± 4.98	46.47 ± 4.99	G	.517	.674	
	CSG^b^	47.16 ± 4.86	49.97 ± 4.81	P	153.507	.001	
	PSG^c^	46.44 ± 5.43	49.12 ± 4.49	G × P	19.450	.001	
	SSG^d^	45.10 ± 3.50	47.68 ± 3.33				
Back strength (kg)	CG^a^	76.80 ± 5.38	77.72 ± 8.34	G	6.567	.001	a < b, c, d
	CSG^b^	74.68 ± 9.56	103.57 ± 9.72	P	506.510	.001	
	PSG^c^	75.63 ± 11.99	103.90 ± 9.84	G × P	53.423	.001	
	SSG^d^	82.38 ± 7.52	105.40 ± 10.33				
40 m sprint (sec)	CG^a^	6.40 ± 0.38	6.38 ± 0.38	G	4.184	.012	b, c, d < a
	CSG^b^	6.12 ± 0.40	5.81 ± 0.35	P	103.792	.001	
	PSG^c^	6.10 ± 0.40	5.80 ± 0.31	G × P	12.305	.001	
	SSG^d^	6.18 ± 0.34	5.69 ± 0.26				
Illinois agility test (sec)	CG^a^	16.81 ± 0.69	16.76 ± 0.69	G	.456	.715	
	CSG^b^	17.27 ± 0.54	16.80 ± 0.50	P	288.253	.001	
	PSG^c^	16.97 ± 0.81	16.57 ± 0.77	G × P	23.408	.001	
	SSG^d^	16.95 ± 0.65	16.52 ± 0.56				
Standing long jump (m)	CG^a^	2.39 ± 0.29	2.39 ± 0.28	G	.033	.992	
	CSG^b^	2.38 ± 0.16	2.44 ± 0.14	P	117.120	.001	
	PSG^c^	2.38 ± 0.19	2.44 ± 0.17	G × P	13.740	.001	
	SSG^d^	2.38 ± 0.17	2.43 ± 0.16				

CG^a^, control group; CSG^b^, compound set group; PSG^c^, pyramid set group; SSG^d^, super set group; G, group; P, period; G × P, Group × Period.

### 3.3 1RM of bench press, deadlift, and squat

The evaluation of the 1RM for bench press, deadlift, and squat among resistance exercise set compositions (Table 5) demonstrated significant interactions between groups and time for these exercises. Post-hoc tests revealed significant differences in bench press (*F = 17.024*, *p = .001*, ηp^2^ = 0.374), deadlift (*F = 23.523*, *p = .001*, ηp^2^ = 0.447), and squat (*F = 19.170*, *p = .001*, ηp^2^ = 0.393) among the groups. Notably, there were significant increases in bench press, deadlift, and squat 1RM in CSG, PSG, and SSG, with no significant differences among the exercise groups.

**TABLE 5:** Changes in 1RM according to the resistance training structure.

Variables	Group	Pre	Post		*F*	*p*	Tukey
Bench press (kg)	CG^a^	44.00 ± 2.42	44.50 ± 5.75	G	7.624	.001	a < b, c, d
	CSG^b^	43.75 ± 5.58	58.22 ± 5.96	P	895.451	.001	
	PSG^c^	43.25 ± 5.90	60.75 ± 7.08	G ×P	93.834	.001	
	SSG^d^	50.75 ± 8.17	66.25 ± 9.07				
Deadlift (kg)	CG^a^	62.25 ± 7.68	64.00 ± 7.19	G	7.472	.001	a < b, c, d
	CSG^b^	61.75 ± 6.78	82.25 ± 7.31	P	1283.544	.001	
	PSG^c^	64.25 ± 4.72	87.50 ± 5.40	G ×P	116.608	.001	a < b, c, d
	SSG^d^	65.00 ± 7.45	84.50 ± 7.53				
Squat (kg)	CG^a^	66.00 ± 6.26	67.25 ± 5.46	G	2.929	.047	
	CSG^b^	63.50 ± 4.74	81.00 ± 3.94	P	1185.800	.001	
	PSG^c^	62.50 ± 7.17	83.50 ± 6.99	G ×P	113.326	.001	
	SSG^d^	62.50 ± 3.91	80.50 ± 4.22				

CG^a^, control group; CSG^b^, compound set group; PSG^c^, pyramid set group; SSG^d^, super set group; G, group; P, period; G × P, Group × Period.

### 3.4 Changes in shoulder stability muscle strength

Examination of changes in shoulder stability muscle strength among resistance exercise set compositions (Table 6) revealed significant interactions between groups and time for the relative values of left and right shoulder abduction and adduction muscles. However, there were no significant differences in shoulder abduction/adduction ratios over time or between groups. Post-hoc tests identified significant differences in the relative values of left shoulder abduction (*F = 12.171*, *p = .001*, ηp^2^ = 0.295), left shoulder adduction (*F = 9.244*, *p = .001*, ηp^2^ = 0.242), right shoulder abduction (*F = 12.289*, *p = .001*, ηp^2^ = 0.297), and right shoulder adduction (*F = 16.707*, *p = .001*, ηp^2^ = 0.363) among the groups. Specifically, CSG and PSG exhibited significantly higher relative values of left shoulder abduction compared to CG and SSG, and PSG showed significantly higher relative values of left shoulder adduction and right shoulder abduction compared to CG. Additionally, PSG demonstrated significantly higher relative values of right shoulder abduction compared to the other groups.

**TABLE 6:** Changes in 1RM according to the resistance training structure.

Variables	Group	Pre	Post		*F*	*p*	Tukey
Left shoulder abduction (%BW)	CG^a^	71.10 ± 9.87	85.50 ± 10.70	G	4.216	.012	a, d < b, c
	CSG^b^	68.10 ± 9.42	103.50 ± 9.10	P	928.081	.001	
	PSG^c^	67.20 ± 6.02	112.10 ± 12.18	G × P	42.933	.001	
	SSG^d^	62.20 ± 12.77	88.50 ± 13.19				
Left shoulder adduction (%BW)	CG^a^	80.00 ± 9.20	97.20 ± 10.79	G	2.324	.091	a < c
	CSG^b^	73.00 ± 14.28	113.50 ± 15.47	P	785.340	.001	
	PSG^c^	70.10 ± 7.00	124.40 ± 9.92	G × P	33.682	.001	
	SSG^d^	67.10 ± 12.38	103.10 ± 12.90				
Right shoulder abduction (%BW)	CG^a^	84.00 ± 11.71	97.00 ± 10.80	G	4.331	.010	a < c
	CSG^b^	78.20 ± 8.08	112.60 ± 12.23	P	808.685	.001	
	PSG^c^	78.60 ± 10.53	126.70 ± 15.20	G × P	44.913	.001	
	SSG^d^	72.50 ± 9.12	100.60 ± 9.45				
Right shoulder adduction (%BW)	CG^a^	87.30 ± 11.18	106.10 ± 12.32	G	5.167	.005	a, b, d < c
	CSG^b^	80.00 ± 12.66	120.40 ± 12.27	P	1783.348	.001	
	PSG^c^	84.60 ± 10.16	142.20 ± 14.26	G × P	79.879	.001	
	SSG^d^	79.30 ± 9.02	113.90 ± 8.40				
Shoulder abduction left/right ratio (%)	CG^a^	85.61 ± 15.19	88.57 ± 11.17	G	.125	.945	
	CSG^b^	86.85 ± 7.78	92.35 ± 7.04	P	9.860	.003	
	PSG^c^	86.08 ± 11.43	89.24 ± 10.38	G × P	.344	.794	
	SSG^d^	85.74 ± 11.54	88.31 ± 13.79				
Shoulder adduction left/right ratio (%)	CG^a^	93.54 ± 20.98	93.35 ± 20.59	G	.732	.540	
	CSG^b^	91.96 ± 16.19	94.27 ± 10.40	P	4.571	.039	
	PSG^c^	83.94 ± 11.72	87.96 ± 9.06	G × P	.82	.487	
	SSG^d^	84.84 ± 13.65	90.57 ± 9.47		8		

CG^a^, control group; CSG^b^, compound set group; PSG^c^, pyramid set group; SSG^d^, super set group; G, group; P, period; G × P, Group × Period; %BW, percent body weight.

### 3.5 Changes in knee stability muscle strength

Analysis of changes in knee stability and muscle strength among resistance exercise set compositions (Table 7) indicated significant interactions between groups and time for the relative values of left and right knee flexion and extension muscles. However, there were no significant differences in knee flexion/extension ratios between groups or over time. Post-hoc tests revealed significant differences in the relative values of left knee flexion (*F = 7.169*, *p = .001*, ηp^2^ = 0.196), left knee extension (*F = 6.471*, *p = .00*, ηp^2^ = 0.179), right knee flexion (*F = 6.576*, *p = .001*, ηp^2^ = 0.182), and right knee extension (*F = 8.191*, *p = .001*, ηp^2^ = 0.182) among the groups. Notably, CSG and PSG exhibited significantly higher relative values of left knee flexion compared to CG, while no significant differences were observed in left knee extension.

**TABLE 7:** Changes in isokinetic knee (60°/sec) muscle strength according to the resistance training structure.

Variables	Group	Pre	Post		*F*	*p*	Tukey
Left knee flexor (%BW)	CG^a^	145.50 ± 29.15	156.50 ± 28.06	G	1.585	.210	a < b, c
	CSG^b^	151.90 ± 21.40	186.90 ± 22.83	P	1877.928	.001	
	PSG^c^	136.90 ± 11.07	199.70 ± 14.35	G ×P	217.117	.001	
	SSG^d^	146.10 ± 23.43	168.40 ± 23.23				
Left knee extensor (%BW)	CG^a^	235.70 ± 31.07	255.50 ± 31.77	G	2.710	.059	a < d
	CSG^b^	250.50 ± 19.43	291.80 ± 27.08	P	757.370	.001	
	PSG^c^	238.30 ± 37.81	288.00 ± 36.23	G × P	45.822	.001	
	SSG^d^	248.00 ± 30.42	323.10 ± 40.85				
Right knee flexor (%BW)	CG^a^	156.10 ± 25.48	164.00 ± 25.12	G	1.107	.359	a, d < c
	CSG^b^	148.30 ± 13.31	180.20 ± 14.44	P	916.839	.001	
	PSG^c^	142.40 ± 23.41	201.90 ± 27.86	G × P	113.398	.001	
	SSG^d^	144.80 ± 14.21	168.40 ± 11.56				
Right knee extensor (%BW)	CG^a^	248.90 ± 22.55	266.20 ± 25.57	G	2.333	.090	a, b < d
	CSG^b^	232.70 ± 14.59	273.00 ± 16.29	P	1163.516	.001	
	PSG^c^	245.90 ± 33.27	295.50 ± 32.40	G × P	79.506	.001	
	SSG^d^	243.70 ± 34.83	318.50 ± 27.86				
Knee flexor left/right ratio (%)	CG^a^	92.69 ± 6.15	94.98 ± 5.45	G	1.002	.403	
	CSG^b^	102.43 ± 12.01	103.80 ± 11.16	P	2.082	.158	
	PSG^c^	98.25 ± 16.06	100.12 ± 11.88	G × P	1.282	.295	
	SSG^d^	101.72 ± 19.25	100.37 ± 14.13				
Knee extensor left/right ratio (%)	CG^a^	94.58 ± 9.18	96.00 ± 9.21	G	1.671	.191	
	CSG^b^	107.80 ± 7.68	106.97 ± 8.70	P	.094	.761	
	PSG^c^	97.52 ± 15.31	97.73 ± 9.98	G × P	.601	.618	
	SSG^d^	104.22 ± 23.48	102.29 ± 17.69				

CG^a^, control group; CSG^b^, compound set group; PSG^c^, pyramid set group; SSG^d^, super set group; G, group; P, period; G × P, Group × Period; %BW, percent body weight.

### 3.6 Core muscle biomechanical properties

The biomechanical parameters of the rectus abdominis (RA) muscle demonstrated statistically significant changes (*P* < 0.05) across all training groups after 8 weeks of intervention, including frequency, stiffness, decrement, relaxation, and creep. However, no significant differences were observed in the changes of these parameters between the different training groups (*P* > 0.05). For the external oblique (EO) muscle, PSG exhibited significantly greater improvements in stiffness compared to the other three groups (F = 51.310, *P* < 0.01, ηp^2^ = 0.182), while no significant differences were found among the other groups (*P* > 0.05). Regarding frequency, the three training groups showed significantly higher values than the CG (F = 23.240, *P* < 0.05, ηp^2^ = 0.436), but no significant differences were observed among the training groups (*P* > 0.05). No significant differences were detected among the groups for parameters such as decrement, relaxation, and creep (*P* > 0.05) (Table 8).

**TABLE 8:** Change muscle mechanical properties according to the resistance training structure.

Variables	Group	Pre	Post		*F*	*p*	Tukey
*Rectus Abdominis* (*RA*)							
Frequency	CG^a^	12.40 ± 0.35	12.43 ± 0.36	G	.816	.496	
	CSG^b^	12.36 ± 0.24	12.64 ± 0.21	P	23.240	.001	
	PSG^c^	12.43 ± 0.31	12.70 ± 0.29	G × P	.112	.953	
	SSG^d^	12.42 ± 0.28	12.64 ± 0.27				
Decrement	CG^a^	0.84 ± 0.04	0.76 ± 0.04	G	2.710	.729	
	CSG^b^	0.86 ± 0.04	0.74 ± 0.04	P	12501.235	.001	
	PSG^c^	0.86 ± 0.04	0.73 ± 0.04	G × P	.436	.001	
	SSG^d^	0.85 ± 0.04	0.72 ± 0.04				
Stiffness	CG^a^	166.70 ± 8.92	168.54 ± 8.73	G	1.107	.276	
	CSG^b^	173.63 ± 8.63	165.15 ± 8.52	P	11681.529	.001	
	PSG^c^	170.68 ± 8.70	158.81 ± 8.20	G × P	3935.196	.001	
	SSG^d^	168.76 ± 8.85	154.80 ± 8.18				
Creep	CG^a^	1.26 ± 0.02	1.25 ± 0.01	G	.333	.801	
	CSG^b^	1.26 ± 0.02	1.24 ± 0.05	P	1.403	.003	
	PSG^c^	1.26 ± 0.02	1.22 ± 0.06	G × P	.343	.795	
	SSG^d^	1.26 ± 0.02	1.24 ± 0.05				
Relaxation	CG^a^	24.81 ± 0.47	24.85 ± 0.47	G	.755	.527	
	CSG^b^	24.76 ± 0.37	24.59 ± 0.37	P	37.462	.001	
	PSG^c^	24.79 ± 0.44	24.62 ± 0.44	G × P	.591	.625	
	SSG^d^	24.80 ± 0.46	24.73 ± 0.46				
*External Oblique* (*EO*)							
Frequency	CG^a^	11.73 ± 0.24	11.72 ± 0.14	G	14.055	.013	a > b, c, d
	CSG^b^	11.62 ± 0.21	11.94 ± 0.20	P	37.461	.001	
	PSG^c^	11.57 ± 0.23	12.25 ± 0.22	G × P	37.252	.021	
	SSG^d^	11.70 ± 0.25	11.97 ± 0.26				
Decrement	CG^a^	1.18 ± 0.08	1.19 ± 0.08	G	.495	.246	
	CSG^b^	1.23 ± 0.05	1.21 ± 0.06	P	1.778	.191	
	PSG^c^	1.91 ± 0.12	1.92 ± 0.09	G × P	.889	.456	
	SSG^d^	1.21 ± 0.06	1.19 ± 0.05				
Stiffness	CG^a^	150.80 ± 4.64	148.80 ± 4.64	G	3.665	.001	c < a, b, d
	CSG^b^	149.40 ± 4.95	147.40 ± 4.95	P	51.310	.001	
	PSG^c^	149.70 ± 5.46	137.70 ± 5.46	G × P	18.742	.021	
	SSG^d^	150.70 ± 6.46	149.60 ± 5.30				
Creep	CG^a^	1.36 ± 0.03	1.36 ± 0.03	G	.573	.636	
	CSG^b^	1.35 ± 0.03	1.36 ± 0.03	P	196.000	.001	
	PSG^c^	1.36 ± 0.03	1.35 ± 0.03	G × P	262.667	.021	
	SSG^d^	1.36 ± 0.03	1.33 ± 0.03				
Relaxation	CG^a^	25.01 ± 0.24	24.53 ± 0.24	G	.448	.720	
	CSG^b^	25.07 ± 0.27	24.60 ± 0.27	P	87349.568	.001	
	PSG^c^	25.01 ± 0.29	24.54 ± 0.29	G × P	31.378	.001	
	SSG^d^	25.12 ± 0.25	24.65 ± 0.25				

CG^a^, Control group; CSG^b^, Compound set group; PSG^c^, Pyramid set group; SSG^d^, Super set group; G, group; P, period; G × P, Group × Period; %BW, percent body weight.

## 4 Discussion

The array of effective resistance training set combinations has expanded considerably over time, reflecting the diverse needs and preferences of contemporary individuals regarding exercise goals, time constraints, and training environments. Despite this proliferation, controversy persists regarding the optimal exercise set configurations for maximizing desired outcomes. Consequently, this study aimed to elucidate the effects of various resistance training set combinations, including compound sets, pyramid sets, and super sets, on body composition, fundamental physical fitness, and isokinetic muscle function of the shoulders and knees among healthy adults.

After an 8-week resistance training program with varied set combinations, the results demonstrated no statistically significant differences in all body composition indicators (weight, skeletal muscle mass, fat mass, body mass index, body fat percentage) compared to the control group. This finding contradicts previous research utilizing pyramid set configurations, which exhibited a decrease in body fat percentage post-exercise (Sayyah et al., 2021). However, it is essential to note that the absence of significant inter-group differences in this study may be attributable to the lack of dietary control for the subjects and the focus of the exercise protocol on analyzing the effects of non-aerobic exercise set combinations, which are believed to exert minimal impact on body composition (Willis et al., 2012; Benito et al., 2020).

The analysis of changes in fundamental physical fitness after the 8-week resistance training program revealed improvements in abdominal strength and 40-m sprint capability in all training groups compared to the control group, although the differences between each training group were not statistically significant. These findings are consistent with prior studies on the effects of different set combinations over 8–12 weeks, which reported significant increases in strength for compound sets, pyramid sets, and super sets (Prasetyo and Nasrulloh, 2017; Merrigan et al., 2019; Fink et al., 2021). Additionally, the increase in lower limb strength is closely associated with enhanced sprint performance (Delecluse, 1997; Seitz et al., 2014). However, the results of this study did not directly investigate the potential influence of neuromuscular junction factors on strength gains, which have been implicated in previous research (Aagaard et al., 2002; Folland and Williams, 2007). Consequently, future research focusing specifically on neuromuscular junction factors and their relationship with resistance training set combinations is warranted.

Comparative analysis of the differences in 1RM (one-repetition maximum) for bench press, deadlift, and squat among resistance training set combinations revealed significant increases in 1RM for all exercises compared to the control group, with no statistically significant differences between each training group. These findings are in line with previous research on 12-week set combinations, which reported significant increases in squat and bench press 1RM for compound sets and super sets (Hadi et al., 2018; Merrigan et al., 2019), respectively, while the pyramid set group exhibited significant increases in 1RM, but with no significant difference in strength gains compared to the drop set group (Angleri et al., 2017). In the 8-week resistance training program conducted in this study, free weight exercises involving technical requirements were considered closed kinetic chain exercises, which may have facilitated the development of muscular proprioception, thereby enhancing balance and coordination (Myhre, 2010; Wilson et al., 2018). However, despite the lack of observed differences between set combinations in this study, it can be inferred that compound sets, pyramid sets, and super sets are suitable for increasing 1RM demands, especially for novice individuals lacking exercise experience (Krieger, 2010; Schoenfeld et al., 2014).

The analysis of changes in isokinetic muscle function demonstrated significant improvements in shoulder and knee isokinetic muscle function in the pyramid set group compared to the control group. These results are consistent with previous research findings, indicating significant increases in muscle strength at 60°/sec after 8 weeks of upper limb resistance training (Alegre et al., 2015) and significant increases in knee flexor and extensor muscle strength after 12 weeks of resistance training in men (Teng et al., 2008; Sung et al., 2016). The progressive overload principle and sufficient rest intervals between sets, inherent in the pyramid set structure, may contribute to improving isokinetic muscle function (Franco et al., 2021; Mahjur and Norasteh, 2021).

Our findings diverge to some extent from previous research investigating the mechanical properties of muscles. Pintar et al. (2009) discovered that traditional isolated abdominal exercises did not significantly enhance the biomechanical performance of the abdominal muscles, while another study demonstrated that comprehensive core training could indeed increase abdominal wall activity and stability (Luo et al., 2022). This discrepancy may stem from differences in the training modalities and measurement methods employed across studies. Our research revealed that sustained pyramidal set training is advantageous for improving the stiffness and vibration frequency of the external oblique muscles, which aligns with the findings of Sundstrup et al. (2012), who reported that targeted training for the lateral abdominal muscles improved the biomechanical characteristics of those specific muscle groups. As a crucial component of the core musculature, optimal biomechanical performance of the external oblique muscles contributes to maintaining dynamic spinal stability. Notably, we did not observe significant differences in rectus abdominis biomechanical properties across different training modalities, which could be attributed to variations in training volume. Previous research has indicated that enhancing the biomechanical performance of the rectus abdominis requires a higher training volume (Lee, 2021).

However, some limitations must be acknowledged. Firstly, the study participants were restricted to a specific region, which may affect the generalizability and applicability of the study results to broader populations. Secondly, psychological factors influencing the study participants’ training performance and results, such as motivation, perceived exertion, and adherence, were not fully controlled, which could potentially impact the observed outcomes. Additionally, despite efforts to control the dietary and physiological phenomena of the study participants, some degree of uncertainty remains, which may impact the study results. For instance, variations in macronutrient intake, hydration levels, and sleep patterns could influence recovery and adaptation processes. Finally, the genetic characteristics of the study participants, which may influence their responses to training and results, were not considered in this study. Future investigations should aim to account for these factors to enhance the robustness and generalizability of the findings.

## 5 Conclusion

The findings of this study indicate that an 8-week resistance training program, comprising distinct combinations of training set configurations, yielded enhancements in fundamental fitness measures and 1RM performances. Notably, the pyramidal set training regimen exhibited pronounced benefits, enhancing isometric functional performance and core muscle biomechanical properties. However, further comparative and validation studies are warranted to elucidate the differential effects of various resistance training set configurations while accounting for covariates such as age, gender, and athletic proficiency. These rigorous research endeavors will provide a robust empirical foundation for tailoring resistance training interventions to specific goals and target populations.

## Data Availability

The raw data supporting the conclusions of this article will be made available by the authors, without undue reservation.
